# Mutational convergence acts as a major player in adaptive parallel evolution of *Shigella* spp.

**DOI:** 10.1038/s41598-019-39810-1

**Published:** 2019-03-01

**Authors:** Achsah K. Thomas, Sruthy Preetha, Anjana Omanakuttan, Lakkaraju Vidyullata, Anjaly Ashokan, Vyshakh Rajachandran, Sujay Chattopadhyay

**Affiliations:** School of Biotechnology, Amrita Vishwa Vidyapeetham, Kollam, 690 525 Kerala India

## Abstract

*Shigella* spp., emerging from multiple origins of *Escherichia coli*, poses a significant health threat as a causative agent of bacillary dysentery. While multiple serotypes of four different species have evolved via independent lineages, *Shigella* spp. are designated as a single pathotype, primarily because of their common mode of pathogenesis. Convergent horizontal transfer events have so far been attributed to the commonalities in the evolution of virulence across diverse lineages. However, the role of mutational convergence in such parallel evolution is not yet well understood. Here we have carried out a genome-wide analysis of *Shigella* strains from all four species to detect the core genes (i.e. the ones present in all analyzed strains) acquiring convergent mutations of evolutionarily recent origin. Simulation studies show non-neutral accumulation of these convergent mutations across species, suggesting their adaptive role in the evolution of *Shigella* virulence. *S. dysenteriae* strain 197, representing highly virulent type 1 (*Sd*1) clone, carries excessively high number of core genes with recent convergent mutations compared to other analyzed strains. We propose that this high frequency of adaptive convergence in *S. dysenteriae* strain 197 could be linked to recent re-emergence of the *Sd*1 clone and its increased resistance to antimicrobials.

## Introduction

*Shigella* spp. have been a severe burden for centuries causing major diarrhoeal diseases across the globe – in under-developed, developing as well as in industrialized countries^1–3^. This enterobacterial pathogen remains the most prevalent one among two to five year old children^4^, and shows an increased emergence of antibiotic resistance^5^. In recent years the rate of mortality caused by shigellosis dropped drastically possibly due to an improved treatment against the highly pathogenic species *Shigella dysenteriae*, thereby resulting in the disappearance of associated epidemics^6^. However, the number of shigellosis cases continues to be high enough with 1.3% annual incidence rate in Asian children below 5 years of age^7^. Also, compared to other infamous enterobacterial pathogens such as *Salmonella* spp. and different diarrhoeagenic pathotypes of *E. coli*, *Shigella* spp. as intracellular pathogens can cause infections via about 4 times lower infectious dose^8^.

Presently, there are four known species of *Shigella* – *S. dysenteriae*, *S. flexneri*, *S. boydii* and *S. sonnei*. While *S. boydii* and *S. dysenteriae* have more or less global representation, *S. flexneri* is prevalent in low-income countries and *S. sonnei* is the commonest pathogen in the high-income ones^9–11^. All these *Shigella* spp. have emerged from *E. coli* several times independently across multiple lineages^10^. It is now known that convergent horizontal transfer events led to the acquisition of several key genes by *Shigella* lineages to gain their virulence and adaptability. Some important horizontally acquired regions are one large (>200 kb) virulence plasmid and several pathogenicity islands such as SHI-1, SHI-2, SRL (*Shigella* resistance locus), etc. to enable toxicity, iron sequestration and antibiotic resistance^12,13^. The evolution of *Shigella* spp. from the extremely diverse species *E. coli* to a highly specialized, human-restricted group of pathogens via convergent horizontal gene transfer events and gene losses have been discussed in some detail^10,11^. But the role of mutational convergence in the adaptive parallel evolution of *Shigella* is poorly studied, although our previous studies^14,15^ showed higher number of genes in *Shigella* spp. than in different *E. coli* pathotypes that accumulated convergent structural (or amino acid) mutations in the encoded proteins. Convergent structural mutations are defined as repeated independent (i.e. phylogenetically unlinked) occurrence of mutations at same amino acid positions of the encoded proteins^16^. Such mutations are considered as a strong signature of adaptive evolution, because their recurrence can be expected to be a positive response to specific selection pressures under similar environmental conditions, thereby offering fitness advantage to the respective organisms^17–24^. However, most of these convergent mutations are found to accumulate relatively recently in evolutionary time without much time to get fixed in the population, and are therefore hard to identify^16,25^.

We here performed a comparative genome-wide analysis of 28 *Shigell*a strains from all four species to identify positive selection footprints via accumulation of recent convergent mutations across different lineages. While we found evolutionarily recent mutational convergence as a potentially critical contributor to adaptive parallel evolution of *Shigella* lineages, *S. dysenteriae* strain 197 exhibited an excessively high fraction of genes with convergent mutations, suggesting a distinctly stronger positive selection dynamics compared to other analyzed strains from diverse lineages. Additionally, many of the identified positively selected genes with potential adaptive mutations convergent across lineages may offer possible targets for novel treatment and preventive measures.

## Results

### Clonal and core genome diversity of analyzed strains

We used PATRIC 3.5.26 database (https://www.patricbrc.org/) to select the set of 28 *Shigella* strains for which the disease phenotype information was publicly available. The sequence types (STs) of these strains were identified using multilocus sequence typing (MLST) scheme for *Escherichia*/*Shigella* (https://enterobase.warwick.ac.uk/species/ecoli/allele_st_search). Complete gene sequences of seven housekeeping loci (*adk*, *fumC*, *gyrB*, *icd*, *mdh*, *purA*, *recA*) were considered for this purpose. The analyzed strains were grouped into a total 15 sequence types or STs (Table 1). While two *S. sonnei* strains were representatives of a single clone of ST152, all the strains of both *S. boydii* and *S. dysenteriae* represented unique STs. Of the 20 *S. flexneri* strains, 50% represented ST245. The remaining 10 strains were members of seven different STs, of which three STs were part of ST245 complex itself. In total, there were 14 strains representing ST245 clonal complex in our dataset (Table 1). The average nucleotide diversity (π) of the MLST loci was 0.01 ± 0.002, and the rates of nonsynonymous (dN) and synonymous (dS) changes were 0.002 ± 0.0004 and 0.037 ± 0.009 respectively.

**Table 1:** Analyzed Strains from four *Shigella* spp. ST denotes multilocus sequence type.

*Shigella* spp.	Strain	Disease	Geographical location	Assembly ID	ST	Clonal complex
*S. flexneri*	CCH060	Gastroenteritis	NA	GCA_000267985.1	145	ST243
	CDC 796–83	Dysentery; Food poisoning; Shigellosis	NA	GCA_000193935.2		
	2a str. 301	Dysentery	China	GCA_000006925.2	245	ST245
	2a str. 2457T	Dysentery	NA	GCA_000007405.1		
	K-218	Dysentery; Food poisoning; Shigellosis	NA	GCA_000213675.2		
	2002017	Dysentery	NA	GCA_000022245.1		
	1235–66	Gastroenteritis	NA	GCA_000268065.1		
	2747–71	Dysentery; Food poisoning; Shigellosis	NA	GCA_000213455.2		
	2930–71	Dysentery; Food poisoning; Shigellosis	NA	GCA_000213455.2		
	4343–70	Dysentery; Food poisoning; Shigellosis	NA	GCA_000213475.2		
	K-404	Gastroenteritis	Bangladesh	GCA_000268025.1		
	K-671	Dysentery; Food poisoning; Shigellosis	Bangladesh	GCA_000213435.2		
	2850–71	Gastroenteritis	NA	GCA_000268085.1	628	
	K-227	Dysentery; Food poisoning; Shigellosis	NA	GCA_000213735.2		
	J1713	Dysentery; Food poisoning	NA	GCA_000217895.2	629	
	5 str. 8401	Dysentery	NA	GCA_000013585.1	634	
	K-1770	Gastroenteritis	Bangladesh	GCA_000268245.1	1025	—
	VA-6	Dysentery; Food poisoning; Shigellosis	Bangladesh	GCA_000213695.2		—
	K-315	Gastroenteritis	Bangladesh	GCA_000268165.1	1512	—
	K-272	Dysentery; Food poisoning; Shigellosis	NA	GCA_000213715.2	5283	—
*S. dysenteriae*	197	Dysentery	China	GCA_000012005.1	146	—
	1012	Dysentery	Bangladesh	GCA_000168075.1	288	ST147
	CDC 74–1112	Dysentery; Food poisoning	NA	GCA_000193895.2	252	ST148
*S. boydii*	227	Dysentery	China	GCA_000012025.1	1130	—
	CDC 3083–94	Dysentery	United States	GCA_000020185.1	1129	—
	ATCC 9905	Dysentery; Food poisoning	United States	GCA_000193915.2	1749	—
*S. sonnei*	046	Dysentery	China	GCA_000092525.1	152	ST152
	53 G	Dysentery; Food poisoning	Japan	GCA_000283715.1		

*S. flexneri* 2a str. 301, due to its well-annotated assembly of protein-coding genes, was used as the reference genome for extracting highly homologous copies of genes from the remaining strains based on 95% nucleotide sequence identity and length coverage. We detected a total of 1450 genes present in all 28 analyzed strains and these genes were therefore defined as core. The π value averaged over all these core genes (0.011 ± 0.0001) was equivalent (P = 0.62) to the MLST π value, while dN and dS values were 0.004 ± 0.0001 and 0.035 ± 0.001 respectively, thereby suggesting that the MLST diversity could be well representative of the overall core genome diversity in *Shigella* species.

### Detection of recent convergent mutations in core genes

Here we adopted zonal phylogeny analysis^16,26^ using TimeZone software^27^ to determine evolutionarily recent acquisition of convergent amino acid mutations. An accepted notion is that the synonymous mutations mostly accumulate randomly but at a constant rate for a given gene, and so the rate of synonymous mutations can serve as a molecular clock^28^. This led us to consider, as explained in earlier works^16,26^, that a structural variant (i.e. showing at least one amino acid change when compared to the ancestral sequence) is evolutionarily old if it is represented by multiple alleles which are separated from one another only by synonymous (i.e. structurally silent) mutations. In the evolution of a given gene, zonal phylogeny separates these synonymously differentiated multi-allelic structural variants from the ones that are represented by single alleles. We hypothesize that these mono-allelic structural variants are evolutionarily recent enough that they are yet to accumulate synonymous variations within them.

We found that 141 genes (~10% of all core genes) accumulated recent convergent changes (Supplementary Table 1). It is noteworthy that the convergent mutations at the same positions could be identical changes (termed parallel convergent mutations), or could be different changes (termed coincidental convergent mutations). Of 141 genes, convergent mutations in 59 genes were of only parallel nature, 66 genes with only coincidental ones, and 16 genes with both types of convergent mutations. Unlike coincidental convergence which would most likely be the result of mutation, parallel convergent changes can originate from either recombination or mutation. However, recombination detection analysis showed absence of recombination in these genes thereby confirming the mutational origin of convergence.

**Figure 1:**
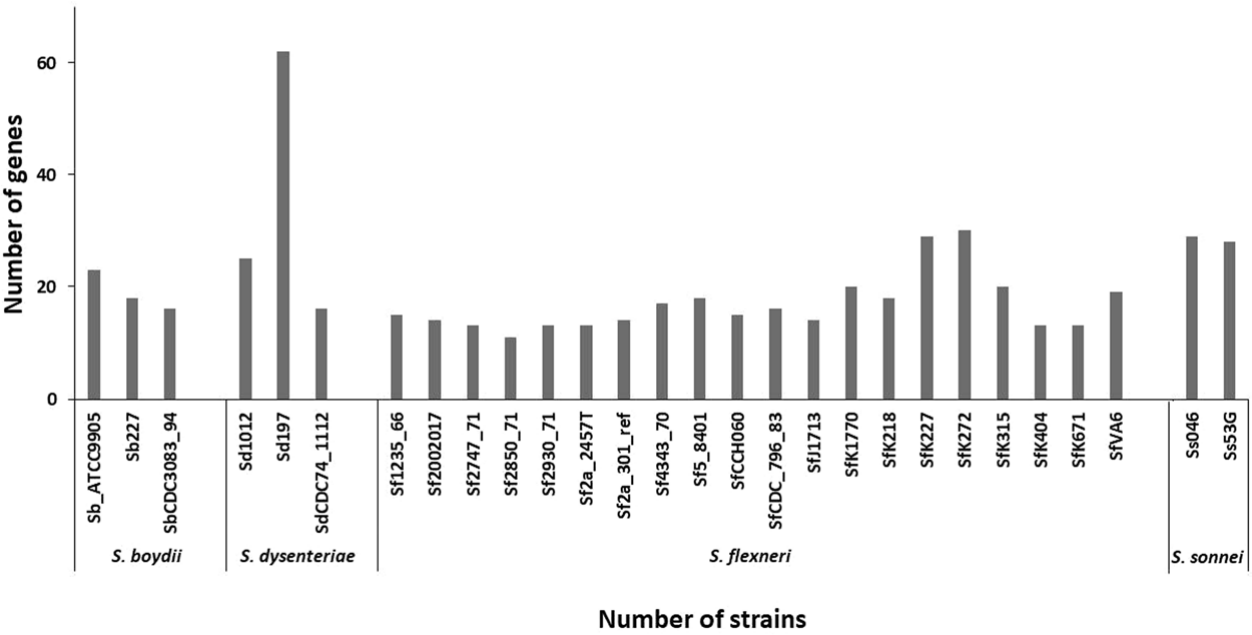
Distribution of recent convergent mutations across *Shigella* species and strains.

As we analyzed the distribution of recent convergent mutations in different *Shigella* species (Fig. 1), we found that except for *S. dysenteriae* strain 197, the number of genes accumulating such mutations ranged from 11 (in *S. flexneri* strain 2850_71) to 30 (again in *S. flexneri* strain K272), the overall average being 18.2 ± 1.1 genes. In contrast, as many as 62 genes (44%) showed acquisition of recent convergent mutations in *S. dysenteriae* strain 197. The three *S. dysenteriae* strains together included 102 genes (72%) with these mutations. Such a high frequency (P < 0.001) in one species (or in one strain to be specific) reflected an extremely skewed distribution of recent mutational convergence across *Shigella* strains.

### Assessment of non-random accumulation of recent convergent mutations

To understand if the skewed distribution of convergent mutations in the core genes is a result of non-random accumulation in response to selection pressures, we assessed the patterns of acquisition of mutational convergence under neutrality. For each of the 141 genes with recent convergent mutations, we ran 10 iterations to perform simulation of mutations. As expected under random probability, we found that, on an average, there was a uniform distribution of recent convergent mutations in 28 simulated sequences, marked A1 through A28 (Supplementary Fig. 1).

**Figure 2:**
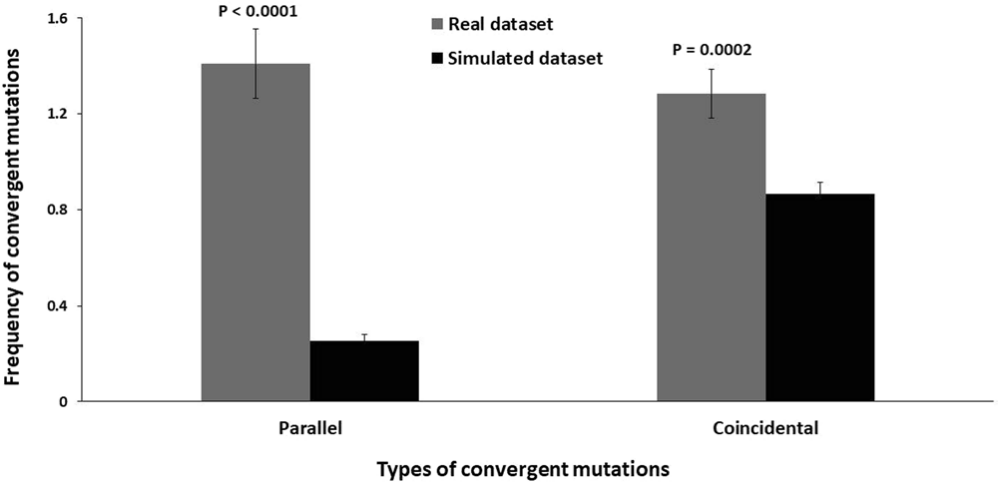
Frequency of recent parallel and coincidental convergent mutations in real and simulated datasets.

Next we compared the frequency of parallel (same mutations) and coincidental (different mutations) convergence in real and simulated datasets. If the mutations accumulate neutrally in the genes, any nucleotide change would be equally probable. Therefore, it is expected that the frequency of coincidental mutations would be much higher than the parallel ones, as evidenced by the simulated frequencies of recent coincidental (0.86 ± 0.05) and parallel (0.26 ± 0.02) convergent mutations (Fig. 2). In sharp contrast, the observed frequencies in real datasets were 1.28 ± 0.10 for the coincidental ones and 1.41 ± 0.14 for the parallel ones, being significantly higher (P < 0.001) than the corresponding values in simulated datasets (Fig. 2). The non-neutral frequency of recent convergent mutations with non-uniform distribution across strains strongly speaks for the accumulation of these mutations in presence of positive selection pressures.

### Co-evolution of genes with recent convergent mutations

**Figure 3:**
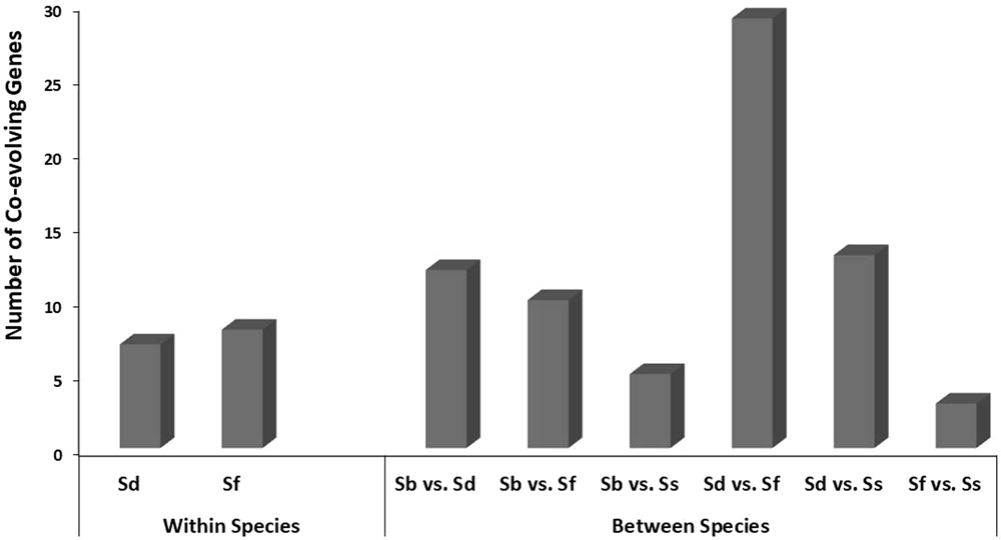
Distribution of co-evolving genes within and between *Shigella* species. Sd: *S. dysenteriae*; Sf: *S. flexneri*; Sb: *S. boydii*; Ss: *S. sonnei*.

We hypothesized that if these 141 candidate genes accumulated adaptive mutational convergence, the sharing of the convergent mutations, i.e. the pairs of structural variants (representing pairs of strains or strain-groups) that accumulated convergent mutations (as exemplified in Supplementary Fig. 2) would also not be random. To test this, we analyzed the co-evolution of genes with recent convergent mutations where each set of genes would be the ones with recent convergence in identical set of strains. We found that there were 87 such co-evolving genes (Supplementary Table 2), of which 29 genes (33%) showed convergence shared exclusively between *S. flexneri* and *S. dysenteriae* (Fig. 3).

As we detected that majority of recent convergent mutations accumulated in *S. dysenteriae*, in strain 197 in particular, it was obvious that *S. dysenteriae* would predominate in the co-evolving set of genes as well. Overall, 61 of 87 (70%) co-evolving genes involved *S. dysenteriae*. Also, out of 28 strains analyzed, 20 were *S. flexneri*. Together, this might be the reason for the high frequency of co-evolving genes among these two species. However, it is noteworthy that as we looked at the presence of co-evolving genes within each species (Fig. 3), we found higher frequency in *S. dysenteriae* (8 genes among only 3 strains from 3 different STs) than in *S. flexneri* (7 genes among 20 strains from 8 different STs). Interestingly, even though we also had 3 *S. boydii* strains from 3 different STs, we did not find any within- species co-evolving genes in *S. boydii*, while two *S. sonnei* strains from ST152 were always clustered (Fig. 3). There were four pairs of strains or (strain-groups) that showed 5 or more co-evolving genes (shown in bold in Supplementary Table 2), i.e. showing independent accumulation of convergent mutations in the two allelic variants of each pair: (a) 9 genes co-evolved in *S. dysenteriae* strain 197 and both the *S. sonnei* strains; (b) 5 genes co-evolved in *S. dysenteriae* strain 197 and two *S. flexneri* strains K1770 and VA6; (c) 9 genes co-evolved in *S. dysenteriae* strain 197 and *S. flexneri* strain K315; (d) 6 genes co-evolved in *S. dysenteriae* strain 197 and common ancestor of *S. boydii* strain ATCC9905 and another *S. dysenteriae* strain 1012. Altogether, selective distribution of co-evolving genes, both within- and between-species, again supports the non-random accumulation of convergent mutations in specific sets of strains potentially under adaptive pressures.

### Analysis of overrepresented functional categories of proteins with recent convergent mutations

**Figure 4:**
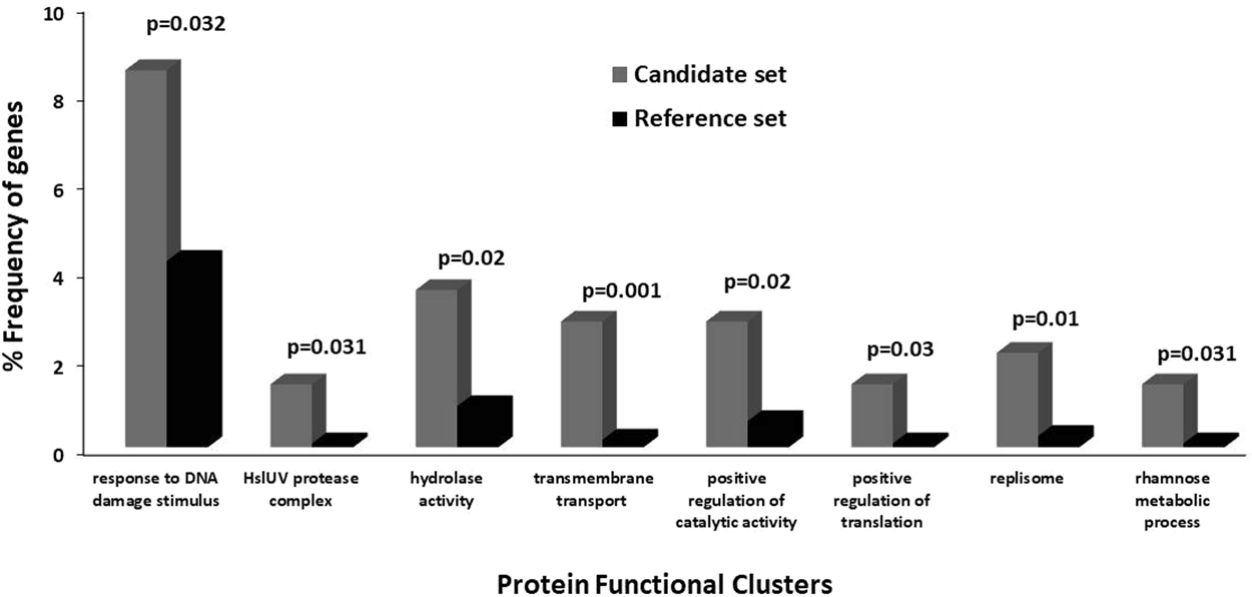
Overrepresented protein functional clusters of candidate positively selected genes with recent convergent mutations.

Of 141 candidate genes encoding protein with recent convergent mutations, 110 genes (78%) were annotated to have defined functions (Supplementary Table 1). It is evident that if positive selection pressures had led to the accumulation of mutational convergence in the core genes, the encoded proteins should not represent a uniform distribution of different functional clusters as expected under random probability. Functional classification of our encoded candidate proteins showed the presence of 19 clusters based on Gene Ontology (GO) categories. As we compared the frequency of candidate genes in each of these functional clusters with the frequency of non-candidate ones (i.e. without any convergent mutations) representing the same cluster across the entire genome, 8 clusters were found to be overrepresented in the candidate set (Fig. 4): response to DNA damage stimulus, hydrolase activity, transmembrane transport, positive regulation of catalytic activity, replisome, HslUV protease complex, positive regulation of translation, and rhamnose metabolic process (in decreasing frequency of represented genes).

Of 87 co-evolving genes, we detected four sets of strain-pairs with 5 or more co-evolving genes (as mentioned in the previous section), with a total of 29 genes (shown in bold in Supplementary Table 2). Notably, in all four sets, one group always happened to be *S. dysenteriae* strain 197. We separately performed the functional enrichment analysis of these 29 genes, of which only 7 genes were annotated as hypothetical proteins. As we detected 8 overrepresented protein clusters in this set, half of them overlapped with the enriched clusters based on the entire candidate set (Supplementary Fig. 3). Functional clusters like glucan metabolic process, DNA replication, epimerase activity and protein phosphatase activity were uniquely overrepresented in this small set of co-evolving genes.

## Discussion

We found 36% of all annotated genes in the reference genome *S. flexneri* 2a str. 301 as core, i.e. present in all analyzed genomes, while 10% of the core genes accumulated convergent structural mutations of evolutionarily recent nature in the encoded protein variants. Such recent emergence of variants via multiple independent mutations at specific positions of selected proteins (termed recent convergent mutations) suggests similar adaptive pressures in different clones competing for survival in the same transient habitats or environmental conditions^25^. Acquisition of two types of convergent mutations – parallel (same changes) and coincidental (different changes) – could be considered as responses to two distinct selection dynamics. The need of any precise functional modification might result in parallel convergence. However, the need to evade host immune pressure or to silence gene expression or function of encoded protein might lead to coincidental changes at critical evolvable sites. Interestingly, the candidate genes showed equivalent exclusive presence of only parallel (in 59 genes) and only coincidental (in 66 genes) types of convergent mutations, with a few (11%) genes having both types of mutations. This suggests co-dominance of two selection dynamics in distinct set of genes.

Significantly higher frequencies of both parallel and coincidental convergence in the real dataset than in the simulated ones indicate acquisition of such mutations potentially in response to positive selection pressures. Depending on the extent of sequence conservation, the proteins can show a highly variable number of mutable sites, while the evolvable sites are always limited. Therefore, in smaller proteins, for instance, in proteins with the length of 100 amino acids or less, as estimated in our previous work on *E. coli*/*Shigella*^14^, the frequency of convergent mutations could be similar to that expected under random probability. However, the average protein size of 272 ± 11 amino acids encoded by our set of candidate 141 genes negates this possibility.

Also, functional enrichment analysis of candidate positively selected genes showed overrepresentation of specific important functional clusters of the encoded proteins. For example, one of these overrepresented clusters is response to DNA damage stimulus. An array of chemical, physical and environmental cues elicit DNA damage in bacteria. Therefore, the organisms need to evolve different well- and under-characterized processes to repair the damaged induced. The type of response/repair mechanism activated depends upon the nature and the extent of DNA damage elicited^29^. One of the candidate genes in this cluster is *nfi*, encoding endonuclease V which is a vital enzyme for deamination repair^30^. An adaptive mutation therein could therefore increase the efficiency of repair mechanism. Also, the role of exoribonucleases in enterobacterial virulence is known, as demonstrated in *Salmonella*^31^, and exodeoxyribonuclease X is one such protein assigned as candidate in this cluster. Recombination protein F (factor F) is responsible for the high frequency of recombination (Hfr) in *Salmonella, Shigella* and *E. coli*^32^. Recombination is necessary to survive the oxidative damage repair within the macrophage, since mutants lacking RecBCD function cannot grow inside macrophages, as shown in *Salmonella*^33^, because of their high sensitivity to macrophage-synthesized oxidative compounds. It is highly likely that adaptive mutation in the recombination protein F (RecF) triggers higher frequency of recombination in *Shigella*. Notably, both the positively selected genes are found to co-evolve via mutational convergence – *nfi* in *S. dysenteriae* strain 197 and *S. flexneri* strains, while *recF* within *S. flexneri* (Supplementary Table 2). Another candidate gene, *tag*, a constitutively expressed gene, encodes 3-methyladenine DNA glycosylase which is crucial for the repair of DNA damage due to alkylation^34^.

Expression of the virulence genes in many cases depends on the pathogen’s ability to simultaneously sense multiple environmental cues (oxygen concentration, pH, etc.)^35^. For this, the RNA binding protein Hfq is an important contributor to both fitness and virulence, while the Hfq mutants become sensitive to host immune system and are found to be even attenuated in animal models^36^. This protein is also found to positively regulate the post-transcriptional cross-talk between the core and accessory genome thereby controlling about one-fifth of all genes in the bacterial pathogens^36^. This protein, as a candidate in the overrepresented cluster of positive regulation of translation and also showing convergent co-evolution in *S. dysenteriae* strain 197 and *S. flexneri* strain K-315 (Supplementary Table 2), potentially indicates critical role of convergent mutations in the virulence evolution of the *Shigella* spp.

Efficient translocation across the entire membrane is essential for the normal metabolism and survival of both pathogenic and non-pathogenic bacteria. Certain proteins that help in such transmembrane transport are shown to be involved in virulence in animal infection models^37,38^. One of these transmembrane transporters is the Tat family of proteins in *E. coli*, *Mycobacterium tuberculosis*, etc. The presence of two twin-arginine translocation proteins, TatA and TatC, as candidates in the overrepresented cluster transmembrane transporter cluster again suggests possible adaptive role of convergent evolution in *Shigella* virulence. Again, both these proteins show co-evolving mutational convergence – TatA within *S. dysenteriae*, while TatC in *S. dysenteriae* strain 197 and *S. flexneri* 5 str. 8401 (Supplementary Table 2). Of all the candidates, 62% genes showed recent convergence being shared by selected groups of strains, indicating adaptive co-evolution of those genes in respective strain-sets. *S. flexneri* and *S. dysenteriae* strains showed predominance in the frequency of co-evolving genes (Fig. 3 and Supplementary Table 2). *S. dysenteriae* is regarded as a perilous *Shigella* species in developing and under-developed countries, while *S. flexneri* is found responsible for the majority of shigellosis cases in resource-poor areas^39^. One explanation for the observed high frequency of co-evolved mutational convergence in these two species might be the commonalities in their pathoadaptive strategies during the outbreaks in developing or under-developed world with similar hygiene challenges and resource-settings. However, higher representation of *S. flexneri* in the list of co-evolving genes might also be attributed, at least in part, to an excess of analyzed strains of this species compared to others.

It is noteworthy that, even though *S. flexneri* and *S. sonnei* appear as the primary causative agents of shigellosis cases across the world^40^, the lowest number of co-evolving genes with recent convergent mutations is noted in these two species (Fig. 3). Such a non-overlap in adaptive convergence could possibly be attributed to different epidemiology of *S. sonnei* and *S. flexneri* pathogenesis, thereby resulting in non-convergent modes of adaptive response to selection pressures at the genetic level. Shigellosis by *S. flexneri* is caused due to the direct attack of the epithelial cell lining of the intestine and its rapid replication in the host cell cytosol^41^. On the other hand, *S. sonnei* has a more naturally advantageous mechanism of infection and dissemination. Firstly, unlike *S. flexneri*, the distribution of *S. sonnei* is not restricted to the countries with poor sanitation facilities, but is extensively reported in industrialized countries^40^. Secondly, certain environmental hosts like *Acanthamoeba castellanii* (*A. castellanii*) helps to provide a natural condition for the patho-adaptation of *S. sonnei*^42^. It is suggested that the growth in this amoebic intracellular niche could have influenced *S. sonnei* to colonize the mammalian phagocytic cells, especially macrophages^43,44^. Since our dataset includes only two *S. sonnei* strains of a single ST in contrast to the diversity of analyzed *S. flexneri* strains, robustness of the observed trend needs to be validated by equivalent number of population-scale datasets from all *Shigella* species.

**Figure 5:**
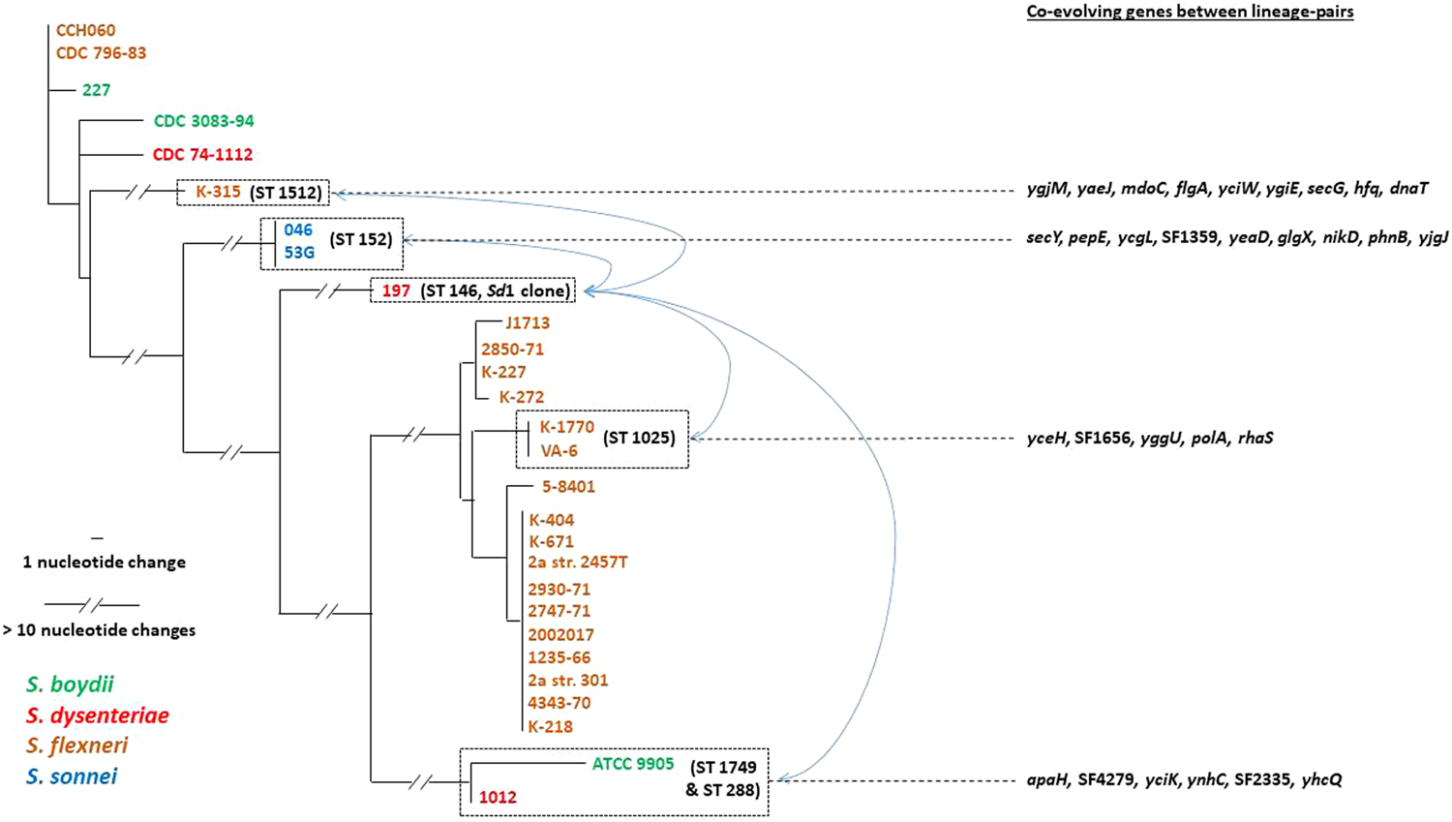
Phylogram of the concatenated sequences of seven MLST housekeeping genes (*adk*, *fumC*, *gyrB*, *icdA*, *mdh*, *purA*, *recA*) showing phylogenetically unlinked nature of *Sd*1 clone (represented by *S. dysenteriae* strain 197) from other *Shigella* lineages as suggested by co-evolving positively selected genes (see Supplementary Table 2). The genes in each set accumulated recent convergent mutations in the corresponding lineage and *S. dysenteriae* strain 197 lineage, suggesting adaptive co-evolution in each of these four lineage-pairs.

On the other hand, although *S. dysenteriae* has been historically associated with large epidemics^45^, presently it is infrequently detected^46^, as is the case for *S. boydii*. However, even in our limited set of analyzed genomes, most remarkable was the scenario of *S. dysenteriae*. It is known that different STs of *S. dysenteriae* emerged through multiple lineages^10,11^, as also suggested by the evolutionary relationships of our analyzed strains based on the concatenated sequences of seven MLST housekeeping genes (Fig. 5). The strain 197 of *S. dysenteriae* showed accumulation of potentially adaptive convergent mutations in almost half of the candidate genes. An array of genes were found to co-evolve via mutational convergence between *S. dysenteriae* ST146 lineage (represented by the strain 197) and (a) *S. sonnei* ST152 lineage, (b) *S. flexneri* ST1025 lineage, (c) *S. flexneri* ST1512 lineage, and (d) the lineage of two closely related clones ST288 (of another *S. dysenteriae*) and ST1749 (of one group of *S. boydii*) (Fig. 5 and Supplementary Table 2). This might indicate that ST146 shares distinctly different adaptive functional trajectories with each of these lineages. However, an alternative possibility is that the functional categories could be common, while different set of proteins are targeted to respond to positive selection pressures. Indeed, functional enrichment analysis of 29 genes representing these four sets of strain-groups having genes co-evolving with ST146 demonstrated some common functional clusters. For instance, parallel evolution of ST146 with ST152, ST1512, and ST288/ST1749 – all accumulated recent convergent mutations in amide transmembrane transporter proteins, as represented by SecY (preprotein translocase subunit), SecG (preprotein translocase subunit), and AaeA (p-hydroxybenzoic acid efflux subunit) respectively. Cellular glucan metabolism was another common process under adaptive evolution in ST146 along with ST152 (glycogen debranching protein GlgX) and ST1512 (glucans biosynthesis protein MdoC). Similarly, nickel ABC transporter ATP-binding protein NikD in ST146 and ST152, and DNA polymerase I protein PolA in ST146 and ST1025 were common representatives of nucleic acid metabolic pathways. Our results suggest that, on one hand, these lineages actually show a convergent nature of adaptive functional trajectories despite the fact that different proteins accumulate those convergent mutations. On the other hand, ST146 lineage of *S. dysenteriae* was exhaustive in response to selection pressures, showing mutational convergence in all of them. Interestingly, the ST146 strain 197 represents *S. dysenteriae* type 1 (*Sd*1), a highly virulent clone causing deadly epidemics^10,47^. We conjecture that the extensive recent adaptive footprints detected in *Sd*1 strain 197 could well be attributed to the recent re-emergence of this clone in association with rapid emergence of antimicrobial resistance as demonstrated by earlier work^47^. However, this association possibility needs to be validated via future work of antibiotic resistance profiling of the analyzed strains. Also, indications of such potentials are premature because of the presence of only one or a few strains in each lineage, and future population-scale studies are warranted to have a clearer understanding of adaptive mutational convergence in the parallel evolution and virulence of *Shigella* lineages.

Altogether, our comparative genomic study suggests that convergent mutations in core genes play an important role in adaptive parallel evolution of *Shigella* lineages. We believe that the information of candidate genes with naturally occurring potential adaptive mutations in different *Shigella* species and strains, along with the co-evolving genes between specific strains/clonal groups will contribute as a valuable resource for future functional studies. This will allow a systematically targeted approach to identify the functions of genes and mutations in relation of physiology, virulence, or drug resistance.

## Materials and Methods

### Analysis of MLST and core genes and detection of recent mutational convergence

Extraction of MLST and other core genes, followed by the analyses of their nucleotide diversity and phylogenetic relationships to detect genes with recent convergent mutations were performed using TimeZone software^27^. A threshold value of 95% was used for both nucleotide sequence identity and length coverage to find orthologs of the reference (*Shigella flexneri* 2a str. 301) genome’s annotated genes in other analyzed genomes. For each gene, maximum-likelihood based zonal phylogeny^26,48^ was constructed to identify convergent structural (amino acid) mutations in evolutionarily recent variants of encoded proteins. Presence of recombination events was assessed in the genes with recent convergent changes using Stepwise program^49^ having MaxChi and PhylPro statistics for detecting recombinants.

### Simulation studies

Each gene was undergone ten rounds of simulation under neutrality using EvolveAGene 3^50^. Each run of simulation generated a random tree topology using the allele of our reference genome as the root sequence, where the branches have equal probability to lead to either a terminal node or an internal node. Observed value of mutation rate, average branch length and average selection on amino acid changes (i.e., dN/dS) were used in the simulation of mutations for the corresponding gene. A constant default modifier value of 1 was set for both sequence-specific and branch-specific selection to simulate the mutations under neutrality. As none of the core genes analyzed had any insertions or deletions in sequences, indels were not allowed in simulated datasets.

### Functional enrichment analysis

Protein functional clusters of the candidate genes were determined by Blast2GO (B2G) platform^51^ via a three-step process of performing BLAST, mapping and annotation based on three GO categories – biological process, molecular function and cellular component. The redundancy of protein-coding genes represented in multiple clusters or GO categories was minimized by a careful selection of functional clusters. While this assignment of candidate genes was considered as a ‘test’ set, functional clusters of the core genes without any convergent mutations were also assigned to serve as the ‘reference’ set in order to detect if the frequency of genes in any of the functional clusters of the ‘test’ set was statistically enriched or overrepresented. Two-tailed Fisher’s Exact Test was used for the test of significance (P < 0.05).

## Supplementary information

Supplementary Figures and Tables

## Data Availability

The datasets that are generated or analyzed during this study but are not included in this published article (and its Supplementary Information files) are available from the corresponding author on reasonable request.
