# Supplementary material for: Mutational convergence acts as a major player in adaptive parallel evolution of Shigella spp
Source: Sci Rep. 2019 Mar 1;9:3252. doi: 10.1038/s41598-019-39810-1 (PMC6397287; doi:10.1038/s41598-019-39810-1)
Supplement: Supplementary file 1 — Supplementary Figures and Tables [file 41598_2019_39810_MOESM1_ESM.pdf]

Mutational convergence acts as a major player in adaptive parallel evolution of *Shigella* spp.

Achsah K Thomas, Sruthy Preetha, Anjana Omanakuttan, Lakkaraju Vidyullata, Anjaly Ashokan, Vyshakh Rajachandran, Sujay Chattopadhyay

School of Biotechnology, Amrita Vishwa Vidyapeetham, Kollam 690 525, Kerala, India

Supplementary Fig. 1. Distribution of recent convergent mutations in the simulated set of 28 strains.

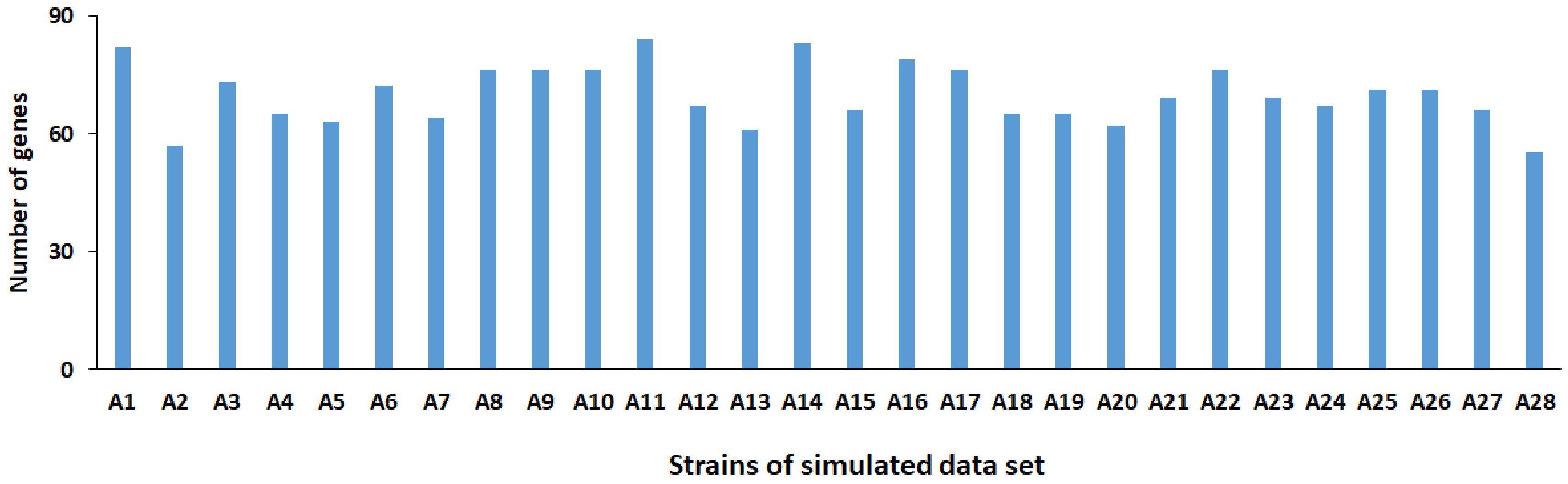

Supplementary Fig. 2. Example of genes co-evolving in strain-pairs via accumulation of convergent mutations. Phylogenetic trees of SecG and Hfq encoding genes show accumulation of nonsynonymous mutations leading to convergent amino acid mutations (at positions 17 and 89 in SecG and Hfq respectively) in identical pair of alleles representing *S. dysenteriae* strain 197 (*Sd197*) and *S. flexneri* strain K_315 (*SfK 315*) in both the genes. Parallel convergent mutations were found in SecG (from valine to isoleucine), while Hfq was targeted by coincidental convergent mutations (alanine to valine in *SfK 315* and alanine to proline in *Sd197*).

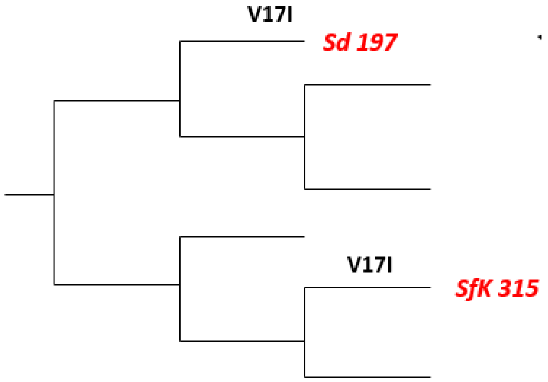

preprotein translocase subunit **SecG**
(intergral membrane component
critical for protein translocation)

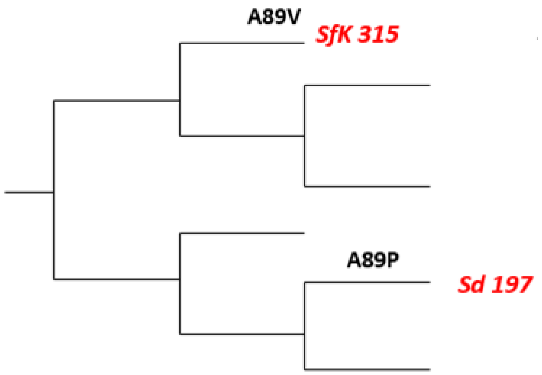

RNA-binding protein **Hfq**
(post-translational regulator critical
for mRNA expression and stability)

Supplementary Fig. 3. Overrepresented protein functional categories of 29 co-evolving positively selected genes with recent convergent mutations.

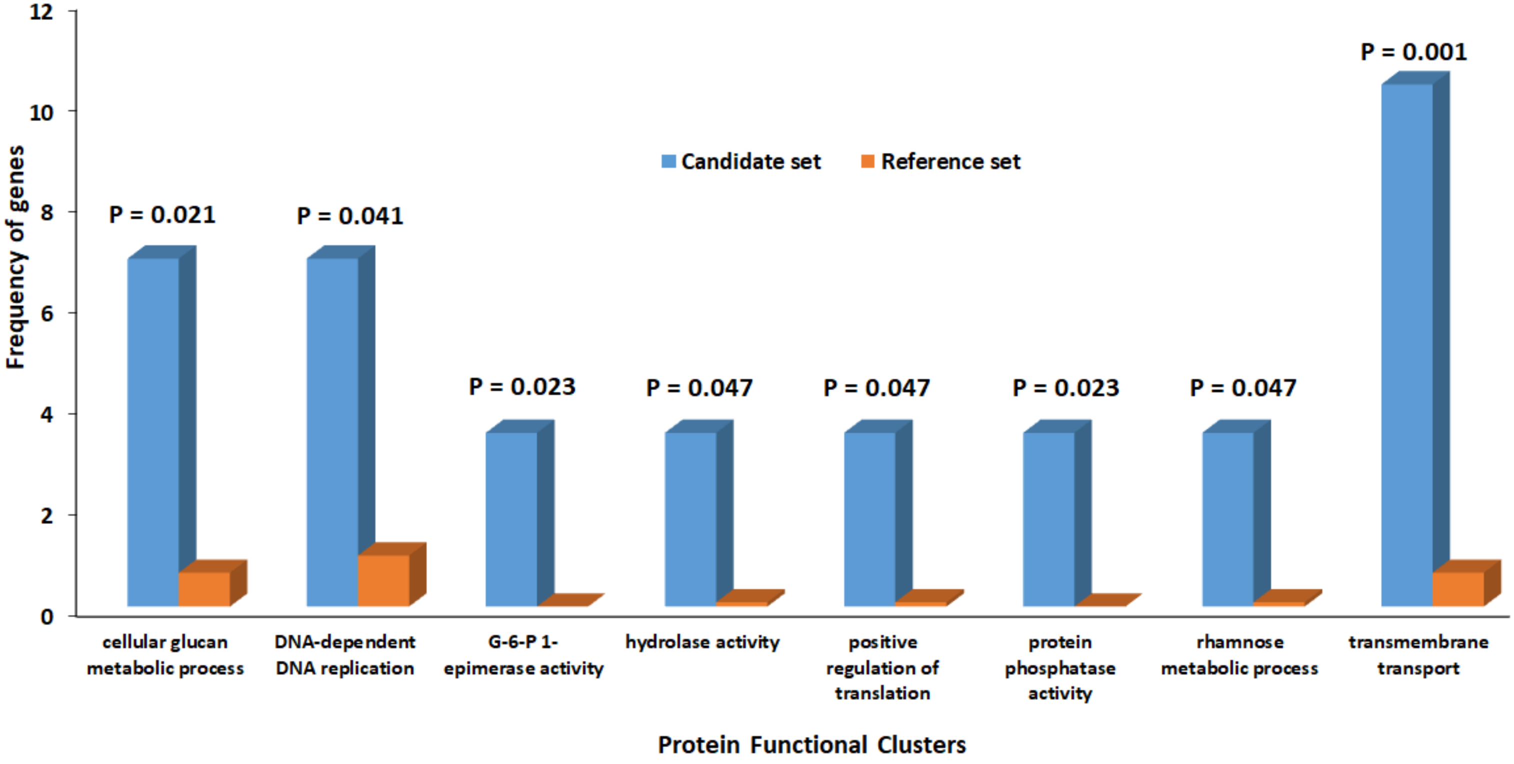

Supplementary Table 1. List of candidate positively selected genes with recent convergent mutations. The name and the coding DNA sequence (CDS) region for each gene was based on the annotation of reference genome *S. flexneri* 2a str. 301.

Gene name	CDS-region	Protein product	Protein length (amino acids)	Type of convergent mutation	Amino acid position of convergent mutations
apaH	49701-50540	diadenosine tetraphosphatase	280	Coincidental	134
djlA	56682-57494	dna-J like membrane chaperone protein	271	Coincidental	151
yadG	140870-141793	ABC transporter ATP-binding protein	308	Coincidental	262
pcnB	149510-150871	poly(A) polymerase	454	Coincidental	209
yaeQ	205235-205777	hypothetical protein	181	Coincidental	152
proC	334081-334887	pyrroline-5-carboxylate reductase	269	Coincidental	146
acrA	420960-422150	multidrug efflux system transporter AcrA	397	Coincidental	214
adk	433700-434341	adenylate kinase	214	Coincidental	201
hemH	434476-435435	Ferrochelatase	320	Coincidental	18
modE	563519-564304	DNA-binding transcriptional regulator modE	262	Coincidental	168
modF	564375-565844	molybdenum transport ATP-binding protein ModF	490	Coincidental	143
SF0798	831551-832756	deoR family transcriptional regulator	402	Coincidental	15
ycdZ	1075147-1075665	hypothetical protein	173	Coincidental	9
mdoG	1086640-1088172	glucan biosynthesis protein G	511	Coincidental	373
yceL	1111882-1113087	multidrug resistance protein MdtH	402	Coincidental	270
ycfB	1193854-1195002	hypothetical protein	383	Coincidental	94
SF1153	1195014-1195472	Phosphohydrolase	153	Coincidental	36
ldcA	1227667-1228560	L,D-carboxypeptidase A	298	Coincidental	282
chaA	1266565-1267662	calcium/sodium:proton antiporter	366	Coincidental	198
yeaD	1478019-1478900	hypothetical protein	294	Coincidental	109
ydjB	1490228-1490884	hypothetical protein	219	Coincidental	82
SF1626	1654194-1655573	arginine/ornithine antiporter	460	Coincidental	130
SF1656	1686922-1687539	electron transport complex protein rnfG	206	Coincidental	47

fdnH	1785962-1786843	formate dehydrogenase-N iron-sulfur subunitbeta	294	Coincidental	216
SF1852	1888470-1888841	hypothetical protein	124	Coincidental	40
SF1855	1889992-1890651	exodeoxyribonuclease X	220	Coincidental	203
yehX	2239279-2240202	ABC transporter ATP-binding protein	308	Coincidental	251
napC	2319660-2320259	cytochrome c-type protein napC	200	Coincidental	9
hisP	2431851-2432621	histidine/lysine/arginine/ornithine ABCtransporter ATP-binding protein	257	Coincidental	129
ubiX	2436171-2436737	3-octaprenyl-4-hydroxybenzoate carboxy-lyase	189	Coincidental	57
ispG	2630132-2631256	4-hydroxy-3-methylbut-2-en-1-yl diphosphatesynthase (flavodoxin)	375	Coincidental	174
yfgA	2631286-2632296	cytoskeletal protein rodZ	337	Coincidental	130
yfhC	2682228-2682761	tRNA-specific adenosine deaminase	178	Coincidental	106
recO	2698958-2699683	DNA repair protein recO	242	Coincidental	123
SF2652	2728828-2729562	outer membrane biogenesis protein bamD	245	Coincidental	172
nrdH	2774616-2774858	glutaredoxin-like protein	81	Coincidental	9
ygdH	2885307-2886668	hypothetical protein	454	Coincidental	285
fucO	2892301-2893446	L-1,2-propanediol oxidoreductase	382	Coincidental	289
yggU	3034665-3034964	hypothetical protein	100	Coincidental	53
mdaB	3165665-3166243	modulator of drug activity B	193	Coincidental	4
yqiB	3169996-3170415	Dehydrogenase	140	Coincidental	21
yraP	3288615-3289187	outer membrane lipoprotein	191	Coincidental	68
yrbI	3334441-3335004	3-deoxy-D-manno-octulosonate 8-phosphatephosphatase KdsC	210	Coincidental	108
yhcP	3374075-3376039	p-hydroxybenzoic acid efflux subunit AaeB	655	Coincidental	512
rpsD	3425441-3426046	30S ribosomal protein S4	202	Coincidental	88
yheM	3444253-3444609	sulfur relay protein TusC	119	Coincidental	33
yhgH	3516128-3516856	hypothetical protein	243	Coincidental	65
gntK	3549715-3550200	Gluconokinase	162	Coincidental	73
gntR	3550381-3551316	gluconate operon transcriptional	312	Coincidental	174

		regulator			
yhiN	3612068-3613264	hypothetical protein	399	Coincidental	135
tag	3684155-3684715	3-methyladenine DNA glycosylase	187	Coincidental	152
yieF	3855500-3856063	hypothetical protein	188	Coincidental	90
tatA	4035042-4035350	twin-arginine translocation protein TatA	103	Coincidental	70
yigU	4035875-4036648	twin-arginine protein translocation systemsubunit TatC	258	Coincidental	253
polA	4059653-4062436	DNA polymerase I	928	Coincidental	215
yihI	4064035-4064541	Der GTPase activator	169	Coincidental	73
rhaS	4111383-4112216	transcriptional activator RhaS	278	Coincidental	5
nfi	4217771-4218439	endonuclease V	223	Coincidental	216
pepE	4247009-4247695	(alpha)-aspartyl dipeptidase	229	Coincidental	46
yjbK	4329718-4330290	Regulator	191	Coincidental	171
pyrI	4415670-4416128	aspartate carbamoyltransferase	44	Coincidental	126
hfq	4505106-4505411	RNA-binding protein Hfq	102	Coincidental	89
SF4353b	4529797-4530108	primosomal replication protein N	104	Coincidental	54;71
dnaT	4567277-4567813	primosomal protein DnaI	179	Coincidental	70
yjjU	4579272-4580342	hypothetical protein	357	Coincidental	259
creC	4602247-4603668	sensory histidine kinase CreC	474	Coincidental	368
yadS	168659-169279	hypothetical protein	207	Parallel	130;138
lpxB	194292-195437	lipid-A-disaccharide synthase	382	Parallel	57;191
yaeJ	205777-206196	hypothetical protein	140	Parallel	120
yaiI	335007-335462	hypothetical protein	152	Parallel	5
SF0390	400715-402136	Transporter	474	Parallel	276
ybiC	778976-780058	Dehydrogenase	361	Parallel	288
SF0767	797244-797708	manganese transport regulator MntR	155	Parallel	9
SF0822	854149-854976	Regulator	276	Parallel	271
SF0823	854979-856025	nucleotide di-P-sugar epimerase or dehydratase	349	Parallel	302
SF0827	860421-861386	HCP oxidoreductase, NADH-dependent	322	Parallel	64
ycbW	992983-993558	hypothetical protein	192	Parallel	93
SF0969	1017987-1018262	Acylphosphatase	92	Parallel	4;10

mdoC	1085092-1086246	glucans biosynthesis protein	385	Parallel	42
flgN	1117178-1117591	flagella synthesis chaperone protein FlgN	138	Parallel	130
ycfS	1157157-1158116	hypothetical protein	320	Parallel	27
potD	1168241-1169284	spermidine/putrescine ABC transportersubstrate-binding protein	348	Parallel	48
ycgL	1211383-1211706	hypothetical protein	108	Parallel	2
narH	1279293-1280828	nitrate reductase 1 subunit beta	512	Parallel	365
SF1249	1301879-1302283	hypothetical protein	135	Parallel	80;114
SF1359	1402922-1403290	tail fiber assembly protein	123	Parallel	114
nadE	1522169-1522993	NAD synthetase	275	Parallel	27
rnt	1706449-1707093	ribonuclease T	215	Parallel	130
cobS	2081822-2082562	adenosylcobinamide-GDP ribazoletransferase	247	Parallel	137
flk	2446061-2447053	flagella biosynthesis regulator	331	Parallel	179
yfeK	2534641-2535012	hypothetical protein	124	Parallel	22
yffB	2579304-2579657	Reductase	118	Parallel	99
dapA	2586942-2587817	4-hydroxy-tetrahydrodipicolinate synthase	292	Parallel	170
hmpA	2670705-2671892	bifunctional nitric oxide	396	Parallel	386
yfiP	2716239-2716958	hypothetical protein	240	Parallel	148
ppnK	2749370-2750245	inorganic polyphosphate/ATP-NAD kinase	292	Parallel	32
recX	2795641-2796138	recombination regulator recX	166	Parallel	130
ygdK	2904976-2905416	CsdA-binding activator	147	Parallel	28
dsbC	2972029-2972736	protein disulfide isomerase	236	Parallel	221
yghB	3149003-3149659	hypothetical protein	219	Parallel	73
ygiE	3175685-3176455	zinc transporter ZupT	257	Parallel	28
ygiM	3190331-3190948	SH3 domain-containing protein	206	Parallel	92
ygjT	3228817-3229776	Transporter	320	Parallel	170
secG	3314359-3314688	preprotein translocase subunit SecG	110	Parallel	17
yhcH	3355112-3355573	hypothetical protein	154	Parallel	7
sspB	3364154-3364648	clpXP protease specificity-enhancing	165	Parallel	160

		factor			
mdh	3371184-3372119	malate dehydrogenase	312	Parallel	190
yhcQ	3376048-3376977	p-hydroxybenzoic acid efflux subunit AaeA	310	Parallel	71
secY	3427152-3428480	preprotein translocase subunit SecY	443	Parallel	143
yheN	3444612-3444995	sulfur transfer complex subunit TusD	128	Parallel	33
glgX	3541997-3543967	glycogen debranching protein	657	Parallel	242
nikD	3587538-3588299	nickel ABC transporter ATP-binding protein NikD	254	Parallel	235
recF	3871535-3872605	recombination protein F	357	Parallel	146
yieP	3950118-3950807	hypothetical protein	230	Parallel	39
yifL	4006227-4006427	outer membrane lipoprotein	67	Parallel	7
SF3908	4028496-4029374	hypothetical protein	293	Parallel	217
fre	4039666-4040364	FMN reductase	233	Parallel	160
hslU	4135496-4136824	ATP-dependent protease ATP-binding subunit HslU	443	Parallel	78
argC	4169756-4170757	N-acetyl-gamma-glutamyl-phosphate reductase	334	Parallel	73
phnB	4282317-4282757	hypothetical protein	147	Parallel	77
tyrB	4321477-4322667	aromatic amino acid aminotransferase	397	Parallel	151
yjgJ	4412402-4412992	hypothetical protein	197	Parallel	139
SF4279	4458425-4459093	FKBP-type peptidylprolyl isomerase	223	Parallel	19
amiB	4500883-4502217	N-acetylmuramoyl-L-alanine amidase	445	Parallel	66
osmY	4578267-4578869	periplasmic protein	201	Parallel	131
yaeF	207081-207902	hypothetical protein	274	Parallel, Coincidental	11
gloB	226932-227684	hydroxyacylglutathione hydrolase	251	Parallel, Coincidental	150;179;220
phoA	253198-254679	alkaline phosphatase	494	Parallel, Coincidental	329
mipB	807630-808361	Transaldolase	244	Parallel, Coincidental	62;64;69

ycaJ	889400-890740	recombination factor protein RarA	447	Parallel, Coincidental	238
yceH	1113918-1114562	hypothetical protein	215	Parallel, Coincidental	42;114
flgA	1117968-1118624	flagellar basal body P-ring biosynthesis protein FlgA	219	Parallel, Coincidental	3;33;110
yciK	1322641-1323396	oxoacyl-(acyl carrier protein) reductase	252	Parallel, Coincidental	87;204
yciW	1343255-1344379	Oxidoreductase	375	Parallel, Coincidental	143;228
ynhC	1743438-1744706	cysteine desulfurase	423	Parallel, Coincidental	23;75;266
SF2335	2382746-2383633	4-deoxy-4-formamido-L-arabinose-phosphoundecaprenol deformylase ArnD	296	Parallel, Coincidental	81;156
truA	2442938-2443747	tRNA pseudouridine synthase A	270	Parallel, Coincidental	93
xseA	2623684-2625051	exodeoxyribonuclease VII large subunit	456	Parallel, Coincidental	125;152;175
yrfC	3490651-3491187	hypothetical protein	179	Parallel, Coincidental	43;86;110
SF3572	3670079-3670351	hypothetical protein	91	Parallel, Coincidental	27;31
lasT	4606492-4607175	RNA methyltransferase	228	Parallel, Coincidental	51

Supplementary Table 2. List of co-evolving positive selected genes with recent convergent mutations. The name and the coding DNA sequence (CDS) region for each gene was based on the annotation of reference genome *S. flexneri* 2a str. 301. Alleles 1 and 2 are the sequence-pairs of strains or strain-groups where the mutations accumulated independently at the same amino acid positions. The rows in bold designate the allele-pairs with 5 or more co-evolving genes, and this set of 29 genes was selected for functional enrichment analysis of co-evolving positively selected genes.

Gene Name	CDS-region	Protein product	Protein length (amino acids)	Functional cluster	Allele 1	Allele 2
apaH	**49701-50540**	**diadenosine tetraphosphatase**	**280**	**nitrogen compound metabolic process**	**197**	**1012, ATCC 9905**
**SF4279**	**4458425-4459093**	**FKBP-type peptidylprolyl isomerase**	**223**	**organic cyclic compound binding**		
yciK	**1322641-1323396**	**oxoacyl-(acyl carrier protein) reductase**	**252**	**binding**		
ynhC	**1743438-1744706**	**cysteine desulfurase**	**423**	**cellular metabolic process**		
**SF2335**	**2382746-2383633**	**4-deoxy-4-formamido-L-arabinose-phosphoundecaprenol deformylase ArnD**	**296**	**hydrolase activity***		
yhcQ	**3376048-3376977**	**p-hydroxybenzoic acid efflux subunit AaeA**	**310**	**transmembrane transport***		
secY	**3427152-3428480**	**preprotein translocase subunit SecY**	**443**	**transmembrane transport***	**197**	**046, 53G**
pepE	**4247009-4247695**	**(alpha)-aspartyl dipeptidase**	**229**	**catalytic activity**		
ycgL	**1211383-1211706**	**hypothetical protein**	**108**	**NA**		

**SF1359**	**1402922-1403290**	**tail fiber assembly protein**	**123**	**phage protein**		
yeaD	**1478019-1478900**	**hypothetical protein**	**294**	**NA**		
glgX	**3541997-3543967**	**glycogen debranching protein**	**657**	**response to DNA damage stimulus***		
nikD	**3587538-3588299**	**nickel ABC transporter ATP-binding protein NikD**	**254**	**organic cyclic compound binding**		
phnB	**4282317-4282757**	**hypothetical protein**	**147**	**NA**		
yjgJ	**4412402-4412992**	**hypothetical protein**	**197**	**NA**		
yceH	**1113918-1114562**	**hypothetical protein**	**215**	**NA**	**197**	**K-1770, VA-6**
**SF1656**	**1686922-1687539**	**electron transport complex protein RnfG**	**206**	**organic cyclic compound binding**		
yggU	**3034665-3034964**	**hypothetical protein**	**100**	**NA**		
polA	**4059653-4062436**	**DNA polymerase I**	**928**	**response to DNA damage stimulus***		
rhaS	**4111383-4112216**	**transcriptional activator RhaS**	**278**	**rhamnose metabolic process***		
ygiM	**3190331-3190948**	**SH3 domain-containing protein**	**206**	**binding**	**197**	**K-315**
yaeJ	**205777-206196**	**hypothetical protein**	**140**	**NA**		
mdoC	**1085092-1086246**	**glucans biosynthesis protein**	**385**	**cellular metabolic process**		
flgA	**1117968-1118624**	**flagellar basal body P-ring biosynthesis protein FlgA**	**219**			
yciW	**1343255-1344379**	**oxidoreductase**	**375**	**catalytic activity**		
ygiE	**3175685-3176455**	**zinc transporter ZupT**	**257**	**transmembrane transport***		
secG	**3314359-3314688**	**preprotein translocase subunit SecG**	**110**	**transmembrane transport***		

hfq	**4505106-4505411**	**RNA-binding protein Hfq**	**102**	**positive regulation of translation***		
dnaT	**4567277-4567813**	**primosomal protein DnaI**	**179**	**replisome***		
yghB	3149003-3149659	hypothetical protein	219	NA	1012	CDC74-1112
tatA	4035042-4035350	twin-arginine translocation protein TatA	103	transmembrane transport*		
phoA	253198-254679	alkaline phosphatase	494	catalytic activity	1012	046, 53G
SF0823	854979-856025	nucleotide di-P-sugar epimerase or dehydratase	349	binding		
ycaJ	889400-890740	recombination factor protein RarA	447	replisome*	1012	4343-70, K-218
SF1852	1888470-1888841	hypothetical protein	124	NA	1012	K-227, K-272
yraP	3288615-3289187	outer membrane lipoprotein	191	periplasmic space		
SF1249	1301879-1302283	hypothetical protein	135	NA	1012	227, CCH060, CDC 796-83
yrbI	3334441-3335004	3-deoxy-D-manno-octulosonate 8-phosphatephosphatase KdsC	188	cellular metabolic process		
mdh	3371184-3372119	malate dehydrogenase	312	cellular metabolic process	197	1012
yhiN	3612068-3613264	hypothetical protein	399	NA		
chaA	1266565-1267662	calcium/sodium:proton antiporter	366	cellular metabolic process	197	1235-66
SF4353b	4529797-4530108	primosomal replication protein N	104	replisome*		

gloB	226932-227684	hydroxyacylglutathione hydrolase	251	hydrolase activity	197	J1713
nrdH	2774616-2774858	glutaredoxin-like protein	81	positive regulation of catalytic activity*		
ygjT	3228817-3229776	transporter	320	transmembrane transport*	197	CDC3083-94
sspB	3364154-3364648	ClpXP protease specificity-enhancing factor	165	positive regulation of translation*		
yieF	3855500-3856063	hypothetical protein	188	NA		
yieP	3950118-3950807	hypothetical protein	230	NA		
hemH	434476-435435	ferrochelatase	320	binding	197	53G
nadE	1522169-1522993	NAD synthetase	275	coenzyme metabolic process		
lpxB	194292-195437	lipid-A-disaccharide synthase	382	carbohydrate derivative metabolic process	197	5 str. 8401
proC	334081-334887	pyrroline-5-carboxylate reductase	269	carboxylic acid biosynthetic process		
yigU	4035875-4036648	twin-arginine protein translocation systemsubunit TatC	258	transmembrane transport*		
ldcA	1227667-1228560	L,D-carboxypeptidase A	298	binding	197	CDC74-1112
yrfC	3490651-3491187	hypothetical protein	179	NA		
yjjU	4579272-4580342	hypothetical protein	357	NA		

hslU	4135496-4136824	ATP-dependent protease ATP-binding subunit HslU	443	HslUV protease complex*	197	K-227, K-272
nfi	4217771-4218439	endonuclease V	223	response to DNA damage stimulus*		
cobS	2081822-2082562	adenosylcobinamide-GDP ribazoletransferase	247	catalytic activity	CDC74-1112	1235_66
SF0798	831551-832756	DeoR family transcriptional regulator	402	transmembrane transport*	K-1770	VA-6
osmY	4578267-4578869	periplasmic protein	201	binding		
yceL	1111882-1113087	multidrug resistance protein MdtH	402	transmembrane transport*	K-1770, VA-6	K-227, K-272
recF	3871535-3872605	recombination protein F	357	response to DNA damage stimulus*		
fdnH	1785962-1786843	formate dehydrogenase-N iron-sulfur subunit beta	294	binding	3083	046, 53G
SF3908	4028496-4029374	hypothetical protein	293	NA		
yehX	2239279-2240202	ABC transporter ATP-binding protein	308	organic cyclic compound binding	K-315	K-227, K-272
gntR	3550381-3551316	gluconate operon transcriptional regulator	312	response to DNA damage stimulus*		
mipB	807630-808361	transaldolase	244	cellular metabolic process	5 str. 8401	1012

SF2652	2728828-2729562	outer membrane biogenesis protein BamD	245	binding		
pcnB	149510-150871	poly(A) polymerase	454	organic cyclic compound binding	5 str. 8401	K-227, K-272
tyrB	4321477-4322667	aromatic amino acid aminotransferase	397			
ycfS	1157157-1158116	hypothetical protein	320	NA	ATCC 9905	046, 53G
yhcH	3355112-3355573	hypothetical protein	154	NA		
yihI	4064035-4064541	Der GTPase activator	169	transmembrane transport*		
yaiI	335007-335462	hypothetical protein	152	NA		
mdoG	1086640-1088172	glucan biosynthesis protein G	511	binding	ATCC 9905	K-315
dapA	2586942-2587817	4-hydroxy-tetrahydrodipicolinate synthase	292	carboxylic acid biosynthetic process		
last	4606492-4607175	RNA methyltransferase	228	organic cyclic compound binding		
ycbW	992983-993558	hypothetical protein	192	NA	ATCC 9905	K-227, K-272
potD	1168241-1169284	spermidine/putrescine ABC transporter substrate-binding protein	348	binding		
hmpA	2670705-2671892	bifunctional nitric oxide	396	organic cyclic compound binding		
ppnK	2749370-2750245	inorganic polyphosphate/ATP-NAD kinase	292	coenzyme metabolic process		
xseA	2623684-2625051	exodeoxyribonuclease VII large subunit	456	catalytic activity	227	CCH060, CDC 796-83
amiB	4500883-4502217	N-acetylmuramoyl-L-alanine amidase	445	hydrolase activity*		

adk	433700-434341	adenylate kinase	214	carbohydrate derivative metabolic process	227	K-671, K-404, 2a str. 2457T, 2930-71, 2747-71, 2002017, 2a str. 301
ybiC	778976-780058	dehydrogenase	361	cellular metabolic process		
yfeK	2534641-2535012	hypothetical protein	124	NA		
